# Sorbitol required for cell growth and ethanol production by *Zymomonas mobilis* under heat, ethanol, and osmotic stresses

**DOI:** 10.1186/1754-6834-6-180

**Published:** 2013-12-05

**Authors:** Kaewta Sootsuwan, Pornthap Thanonkeo, Nawapote Keeratirakha, Sudarat Thanonkeo, Prasit Jaisil, Mamoru Yamada

**Affiliations:** 1 Division of Biotechnology, Faculty of Agro-Industrial Technology, Rajamangala University of Technology Isan, Kalasin Campus, Kalasin 46000, Thailand; 2 Department of Biotechnology, Faculty of Technology, Khon Kaen University, Khon Kaen 40002, Thailand; 3 Fermentation Research Center for Value Added Agricultural Products, Khon Kaen University, Khon Kaen 40002, Thailand; 4 Walai Rukhavej Botanical Research Institute, Mahasarakham University, Mahasarakham 44150, Thailand; 5 Department of Plant Sciences and Agricultural Resources, Faculty of Agriculture, Khon Kaen University, Khon Kaen 40002, Thailand; 6 Department of Biological Chemistry, Faculty of Agriculture, Yamaguchi University, Yamaguchi 753-8515, Japan

**Keywords:** *Zymomonas mobilis*, Glucose-fructose oxidoreductase, Ethanol production, Heat stress, Ethanol stress, Osmotic stress, Fusion-PCR-based construction technique

## Abstract

**Background:**

During ethanol fermentation, the ethanologenic bacterium, *Zymomonas mobilis* may encounter several environmental stresses such as heat, ethanol and osmotic stresses due to high sugar concentration. Although supplementation of the compatible solute sorbitol into culture medium enhances cell growth of *Z. mobilis* under osmotic stress, the protective function of this compound on cell growth and ethanol production by this organism under other stresses such as heat and ethanol has not been described yet. The formation of sorbitol in *Z. mobilis* was carried out by the action of the glucose-fructose oxidoreductase (GFOR) enzyme which is regulated by the *gfo* gene. Therefore, the *gfo* gene in *Z. mobilis* was disrupted by the fusion-PCR-based construction technique in the present study, and the protective function of sorbitol on cell growth, protein synthesis and ethanol production by *Z. mobilis* under heat, ethanol, and osmotic stresses was investigated.

**Results:**

Based on the fusion-PCR-based construction technique, the *gfo* gene in *Z. mobilis* was disrupted. Disruption of the *Z. mobilis gfo* gene resulted in the reduction of cell growth and ethanol production not only under osmotic stress but also under heat and ethanol stresses. Under these stress conditions, the transcription level of *pdc*, *adhA*, and *adhB* genes involved in the pyruvate-to-ethanol (PE) pathway as well as the synthesis of proteins particularly in *Z. mobilis* disruptant strain were decreased compared to those of the parent. These findings suggest that sorbitol plays a crucial role not only on cell growth and ethanol production but also on the protection of cellular proteins from stress responses.

**Conclusion:**

We showed for the first time that supplementation of the compatible solute sorbitol not only promoted cell growth but also increased the ethanol fermentation capability of *Z. mobilis* under heat, ethanol, and osmotic stresses. Although the molecular mechanism involved in tolerance to stress conditions after sorbitol supplementation is still unclear, this research has provided useful information for the development of the effective ethanol fermentation process particularly under environmental conditions with high temperature or high ethanol and sugar concentration conditions.

## Introduction

*Zymomonas mobilis*, the Gram-negative facultative anaerobic bacterium, is unique in employing the Entner Doudoroff (ED) (2-keto-3-deoxy-6-phosphogluconate, KDPG), glyceraldehyde-3-phosphate-to-pyruvate (GP), and pyruvate-to-ethanol (PE) pathway for sugar catabolism and produces ethanol, and carbon dioxide as dominant fermentation products [1]. It generates one mole of ATP per mole of glucose utilized, which is a much lower level than that of the traditional ethanol-producing yeast, *Saccharomyces cerevisiae*. This bacterium thus appears to maintain a high level of glucose flux through these metabolic pathways to compensate its low ATP yield [2], for which large amounts of enzymes in the ED-GP-PE pathway are expressed, constituting 30 to 50% of total soluble proteins of cells [1]. *Z. mobilis* has a relatively compact genome with a small number of genes (approximately 2,000 genes [3]). It possesses an incomplete Embden Meyerhof Parnas (EMP) pathway and an incomplete tricarboxylic acid cycle (TCA cycle) due to a lack of genes for 6-phosphofructokinase, 2-oxoglutarate dehydrogenase complex, and malate dehydrogenase [1-3], but possesses strong activities of the ED-GP pathway [4]. Comparative studies of both laboratory- and pilot-scales on kinetics of batch fermentation of *Z. mobilis* versus a variety of yeast have indicated the suitability of *Z. mobilis* over yeasts due to the following advantages: its higher sugar uptake rate and ethanol yield (97% theoretical maximum yield), lower biomass production, and higher ethanol tolerance. Additionally, it does not require a controlled addition of oxygen during fermentation, it is amenable to genetic manipulation, and most importantly it is generally recognized as safe (GRAS) [1,5].

During fermentation, *Z. mobilis* produces not only ethanol and carbon dioxide but also an alcohol sugar, sorbitol, as a major by product when it is grown in sucrose or mixtures of glucose plus fructose medium [6-8]. Viikari [8] reported that the amount of sorbitol produced is equivalent to as much as 11% of the original carbon source. Only minor amounts of sorbitol are formed from glucose or fructose alone. The formation of sorbitol resulted from the *in vivo* inhibition of fructokinase by glucose. Subsequently, fructose is accumulated and then converted into sorbitol by the action of glucose-fructose oxidoreductase (GFOR), a periplasmic enzyme which comprises 1% of the total soluble protein in *Z. mobilis*[7,9,10]. Previous studies by Loos *et al*. [11] demonstrated that the addition of sorbitol into culture medium promotes the growth of *Z. mobilis* when grown in a high-sugar medium. This finding suggested that sorbitol protected cells from harmful effects caused by high osmotic pressures. However, during ethanol fermentation, *Z. mobilis* may encounter not only high osmotic stress but also heat and ethanol stresses [12,13], which adversely affect the ability of cells to perform efficient and consistent conversion of sugars to ethanol. Although the involvement of sorbitol in osmoprotection has been previously described [11], its protective function on cell growth and ethanol fermentation ability of *Z. mobilis* under heat and ethanol stresses has not been investigated yet. In the present study, we disrupted the *gfo* gene which encoded for GFOR in *Z. mobilis* and the disruptant strain was designated as *Z. mobilis Δgfo*. We showed for the first time that disruption of the *gfo* gene resulted in the reduction of cell growth and ethanol production in *Z. mobilis* under osmotic stress as well as under heat and ethanol stresses. We also demonstrated that the addition of sorbitol not only promoted cell growth but also increased the fermentation capability of *Z. mobilis* under all stress conditions tested. Sorbitol also protected cellular proteins from denaturation under stress conditions.

## Results

### Disruption of the gfo gene in *Z. mobilis*

**Figure 1:**
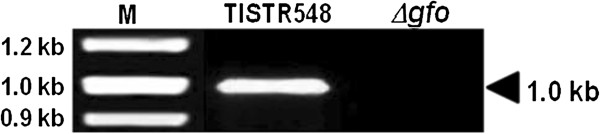
**PCR analysis of the *gfo *gene in *Z. mobilis *parental (TISTR548) and disruptant (*Δgfo*) strains.** The size of the PCR product corresponding to an internal fragment of the *gfo* is indicated on the right. M, indicates the 100 bp DNA ladder.

For the disruption of the *gfo* gene, plasmid pZA-UDGFOR containing the up- and down-region of the *gfo* gene linked with *kan* cassette was transformed into *Z. mobilis*, and kanamycin-resistant transformants were selected. Among the kanamycin-resistant transformants obtained in this study, one isolate designated as *Z. mobilis Δgfo* was chosen for disruption analysis of the *gfo* gene. Disruption of the *gfo* gene in *Z. mobilis Δgfo* was confirmed by PCR using specific primers GFOR-int-5’ and GFOR-int-3’, as described in the Methods section. As shown in Figure 1, a PCR product of approximately 1.0 kb corresponding to the internal fragment of the *gfo* gene was observed in the *Z. mobilis* parental strain, but not in the transformant. The nucleotide sequence of the PCR product from the parental strain showed a high degree of identity (99%) with the *gfo* gene in the *Z. mobilis*, confirming that this amplified DNA fragment was a part of the *gfo* gene in this microorganism (data not shown).

Disruption of the *gfo* gene in *Z. mobilis* was further confirmed by detection of sorbitol concentration in the *Z. mobilis* parental strain and transformant after cultivation in YP medium containing 300 g/L sucrose as a carbon source. The results found that approximately 3.84 g/L of sorbitol was detected in the *Z. mobilis* parental strain. However, the level of sorbitol was not detectable in the transformant. Based on this result and PCR analysis, it was concluded that the *gfo* gene of *Z. mobilis* was disrupted in the transformant strain.

### Effect of stresses on growth of *Z. mobilis* parental and disruptant strains

**Figure 2:**
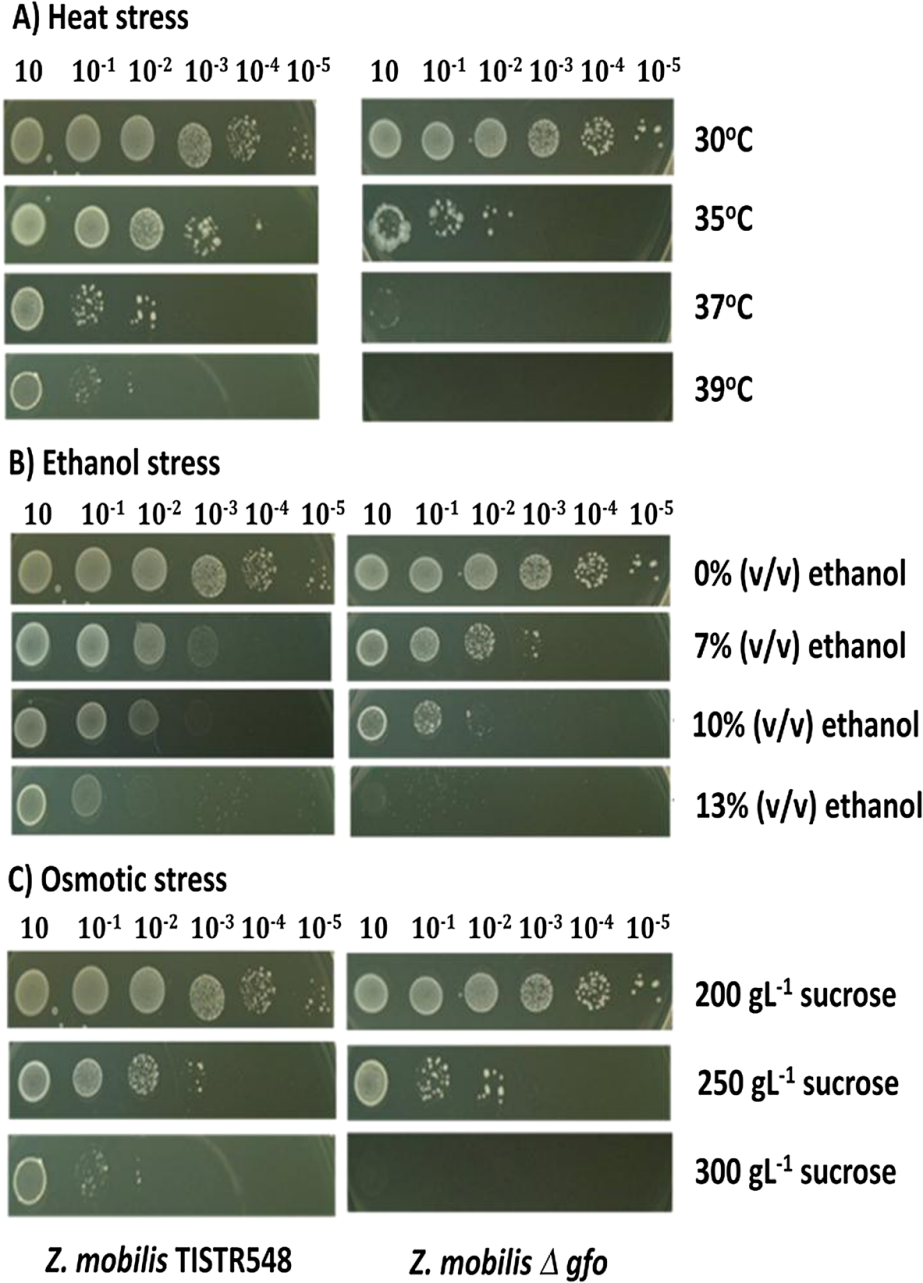
**Effect of heat, ethanol, and osmotic stresses on the growth of *Z. mobilis *TISTR548 and *Z. mobilis Δgfo*. (A)***Z. mobilis* cells were grown in YP medium and incubated at 30, 35, 37, and 39°C or **(B)** in YP medium containing 7, 10, and 13 % (v/v) ethanol, **(C)** or in YP medium containing 200, 250 and 300 g/L sucrose.

The effect of heat, ethanol, and osmotic stresses on the growth of *Z. mobilis* parental and disruptant strains were investigated and the results are illustrated in Figure 2. As shown in Figure 2A, growth of *Z. mobilis* TISTR548 and *Z. mobilis Δgfo* was not significantly different when they were incubated at 30°C. However, when temperatures were shifted from 30°C to 35°C and 37°C, growth of *Z. mobilis Δgfo* was remarkably decreased as compared to that of *Z. mobilis* TISTR548. At 39°C, almost no growth of *Z. mobilis Δgfo* was observed. The effect of ethanol stress on the growth of *Z. mobilis* is shown in Figure 2B. There were no significant differences in the growth of *Z. mobilis* TISTR548 and *Z. mobilis Δgfo* when they were grown on YPG medium containing 0% (v/v) ethanol. However, when ethanol concentration in the medium was increased up to 7% or 10% (v/v), a pronounced decrease in growth of *Z. mobilis Δgfo* was observed. In the YPG medium containing 13% (v/v) ethanol, almost no growth of *Z. mobilis Δgfo* was found. Figure 2C shows the effect of osmotic stress on cell growth of *Z. mobilis* TISTR548 and *Z. mobilis Δgfo*. Growth of *Z. mobilis* TISTR548 and *Z. mobilis Δgfo* was not significantly different when they were grown on YP medium containing 200 g/L sucrose. When the concentration of sucrose in the medium was increased, a drastically decreased growth of *Z. mobilis Δgfo* was observed as compared to that of *Z. mobilis* TISTR548. Almost no growth of *Z. mobilis Δgfo* was detected when it was cultured on YP medium containing 300 g/L sucrose.

The effect of heat, ethanol, and osmotic stresses on the specific growth rate of *Z. mobilis* TISTR548 and *Z. mobilis Δgfo* was also determined and the results are summarized in Table 1. The specific growth rates of *Z. mobilis Δgfo* were lower than those of *Z. mobilis* TISTR548 under all stress conditions tested. When incubation temperatures, ethanol concentrations, or sucrose concentrations increased, the specific growth rates of both *Z. mobilis* TISTR548 and *Z. mobilis Δgfo* were decreased. The lowest specific growth rates were found in *Z. mobilis Δgfo* growing under the extreme stress conditions, (at 39°C or 13% (v/v) ethanol or 300 gL^-1^ sucrose, which were 1.89-, 2.84-, and 2.09-fold lower than those of *Z. mobilis* TISTR548 growing under the same conditions, respectively). These results are similar to those observed in Figure 2. It should be noted from these findings that *Z. mobilis Δgfo* was highly sensitive to heat, ethanol, and osmotic stresses as compared to *Z. mobilis* TISTR548.

**Table 1:** **Specific growth rate of *Z. mobilis *** **TISTR548 and *Z. mobilis Δgfo *** **under heat, ethanol, and osmotic stresses**

**Stress conditions**	**Specific growth rate (h**^**-1**^**)**	
	***Z. mobilis *** **TISTR548**	***Z. mobilis Δgfo***
*Heat stress*		
30°C	0.574 ± 0.27	0.518 ± 0.12
35°C	0.531 ± 0.18	0.310 ± 0.04
37°C	0.287 ± 0.26	0.102 ± 0.20
39°C	0.121 ± 0.22	0.064 ± 0.12
*Ethanol stress*		
0% (v/v) ethanol	0.587 ± 0.24	0.512 ± 0.18
7% (v/v) ethanol	0.417 ± 0.17	0.435 ± 0.27
10% (v/v) ethanol	0.326 ± 0.18	0.290 ± 0.15
13% (v/v) ethanol	0.142 ± 0.20	0.050 ± 0.16
*Osmotic stress*		
200 gL^-1^ sucrose	0.540 ± 0.23	0.502 ± 0.03
250 gL^-1^ sucrose	0.448 ± 0.27	0.349 ± 0.10
300 gL^-1^ sucrose	0.119 ± 0.24	0.057 ± 0.18

### Effect of stresses on ethanol production by *Z. mobilis* parental and disruptant strains

The effect of heat stress on ethanol production by *Z. mobilis* TISTR548 and *Z. mobilis Δgfo* was performed and the results are shown in Table 2. The ethanol concentration produced by *Z. mobilis Δgfo* at 30°C was 57.45±5.03 g/L, which was 1.27-fold lower than that of the parental strain, *Z. mobilis* TISTR548. At 35°C and 37°C, the ethanol concentrations produced by *Z. mobilis Δgfo* were 21.00±0.63 g/L and 17.96±0.42 g/L, respectively, which were 2.34- and 2.33-fold lower than those of *Z. mobilis* TISTR548. Although the ethanol yields from *Z. mobilis* TISTR548 and *Z. mobilis Δgfo* were not much different, the volumetric ethanol productivities of *Z. mobilis* TISTR548 were about 1.51-, 2.28-, and 2.32-fold higher than those of *Z. mobilis Δgfo* at 30°C, 35°C, and 37°C, respectively.

**Table 2:** **Effect of heat, ethanol and osmotic stresses on ethanol production by *Z. mobilis *** **TISTR548 and *Z. mobilis Δgfo***

**Fermentation parameter**	***Z. mobilis *** **TISTR548**			***Z. mobilis Δgfo***		
*Heat stress*	30°C	35°C	37°C	30°C	35°C	37°C
*P* (g L^-1^)	72.69 ± 10.62	49.06 ± 1.46	41.87 ± 4.93	57.45 ± 5.03	21.00 ± 0.63	17.96 ± 0.42
*Qp* (g L^-1^ h^-1^)	1.21 ± 0.18	1.02 ± 0.04	1.16 ± 0.14	0.80 ± 0.07	0.44 ± 0.01	0.50 ± 0.01
*Yps* (g g^-1^)	0.47 ± 0.07	0.46 ± 0.08	0.46 ± 0.03	0.47 ± 0.06	0.40 ± 0.10	0.44 ± 0.09
Time (hours)	60	48	36	72	48	36
*Ethanol stress*	0%	7%	10%	0%	7%	10%
*P* (g L^-1^)	74.60 ± 3.08	40.73 ± 2.68	10.66 ± 2.24	58.03 ± 5.16	29.36 ± 2.24	2.68 ± 0.44
*Qp* (g L^-1^ h^-1^)	1.55 ± 0.20	0.85 ± 0.12	0.22 ± 0.10	0.97 ± 0.12	0.61 ± 0.04	0.07 ± 0.02
*Yps* (g g^-1^)	0.48 ± 0.07	0.44 ± 0.10	0.35 ± 0.05	0.47 ± 0.08	0.37 ± 0.12	0.10 ± 0.03
Time (hours)	48	48	48	60	48	36
*Osmotic stress*	200 g/L	250 g/L	300 g/L	200 g/L	250 g/L	300 g/L
*P* (g L^-1^)	77.67 ± 1.89	69.85 ± 2.44	34.42 ± 1.12	54.12 ± 5.22	45.80 ± 0.45	1.12 ± 0.25
*Qp* (g L^-1^ h^-1^)	1.29 ± 0.03	1.16 ± 0.04	0.57 ± 0.14	0.75 ± 0.07	0.64 ± 0.02	0.02 ± 0.02
*Yps* (g g^-1^)	0.50 ± 0.01	0.51 ± 0.01	0.35 ± 0.05	0.46 ± 0.11	0.44 ± 0.01	0.07 ± 0.03
Time (hours)	60	60	60	72	72	72

*P*, ethanol concentration; *Qp*, volumetric ethanol productivity; *Yps*, ethanol yield**.**

Table 2 also shows the effect of ethanol stress on ethanol production by *Z. mobilis* TISTR548 and *Z. mobilis Δgfo*. The ethanol concentration, volumetric ethanol productivity, and ethanol yield obtained from *Z. mobilis Δgfo* grown in the medium containing 0 to 10% (v/v) ethanol were lower than those from *Z. mobilis* TISTR548. In the medium containing 10% (v/v) ethanol, only 2.68 ± 0.44 g/L ethanol was produced by *Z. mobilis Δgfo*, which was 3.98-fold lower than that of *Z. mobilis* TISTR548 growing in the same condition.

We also tested the effect of osmotic stress on ethanol production by *Z. mobilis* TISTR548 and *Z. mobilis Δgfo* and the results are summarized in Table 2. *Z. mobilis Δgfo* produced 54.12 ± 5.22 and 45.80 ± 0.45 g/L ethanol concentration when cultured in the medium containing 200 and 250 g/L sucrose, which was 1.44- and 1.53-fold lower than that of *Z. mobilis* TISTR548 in the same condition, respectively. In addition, only 1.12±0.25 g/L ethanol concentration was produced by *Z. mobilis Δgfo* when cultured in the medium containing 300 g/L sucrose, which was 30.73-fold lower than that of *Z. mobilis* TISTR548. These results clearly demonstrated that the fermentation activity of the *Z. mobilis* disruptant strain, *Z. mobilis Δgfo*, was highly sensitive to osmotic stress compared to that of the parental strain, *Z. mobilis* TISTR548, as observed under heat and ethanol stresses.

### Real-time RT-PCR analysis of pdc, adhA, and adhB genes under stress conditions

**Figure 3:**
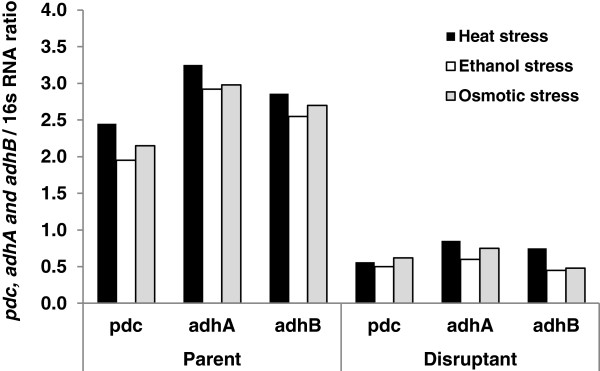
**Expression levels of *pdc*, *adhA*, and *adhB *genes in *Z. mobilis *TISTR548 and *Z. mobilis Δgfo *under heat, ethanol, and osmotic stresses after real-time RT-PCR analysis.** Values presented as the mean and relative expression levels of each gene as described in the Methods section.

The ethanol production capability of *Z. mobilis* disruptant strain as measured by ethanol content was lower than that of the parental strain under all stress conditions tested. This finding led us to the hypothesis that heat, ethanol, and osmotic stresses may suppress the expression of genes involved in the PE pathway. To test this hypothesis, we determined the expression levels of *pdc*, *adhA*, and *adhB* genes in the disruptant strain by real-time RT-PCR using 16 s RNA as an internal control and compared its expression with those in the parental strain under all stress conditions. As shown in Figure 3, the mRNA expression levels of *pdc*, *adhA*, and *adhB* of *Z. mobilis* TISTR548 under heat stress were 4.38-, 3.82-, and 3.81-fold higher than those of *Z. mobilis Δgfo*, respectively. Under ethanol stress, the expression levels of *pdc*, *adhA*, and *adhB* of *Z. mobilis* TISTR548 were 3.90-, 4.87- and 5.67-fold higher than those of *Z. mobilis Δgfo*, respectively. Likewise the expression levels of *pdc*, *adhA*, and *adhB* of *Z. mobilis* TISTR548 under osmotic stress were 3.47-, 3.97-, and 5.63-fold higher than those of *Z. mobilis Δgfo*, respectively. These findings suggested that the expressions of *pdc*, *adhA*, and *adhB* in the *Z. mobilis* parental strain were higher than those in the disruptant strain, which in turn lead to the high ethanol fermentation capability particularly under stress conditions. Due to the suppression of *pdc*, *adhA*, and *adhB* genes by heat, ethanol, and osmotic stresses, the ethanol production by *Z. mobilis Δgfo* was lower than that of the parental strain.

### Protective function of sorbitol on cell growth and ethanol production by *Z. mobilis* parental and disruptant strains

To determine the protective function of sorbitol on cell growth and ethanol fermentation ability by *Z. mobilis* TISTR548 and *Z. mobilis Δgfo* under stress conditions, cells were exposed to heat (37°C), ethanol (10%), or osmotic stress (300 g/L sucrose) in the presence of 50 mM sorbitol. As shown in Table 3, supplementation of sorbitol into the culture medium enhanced cell growth (as specific growth rate) as well as the fermentation capability of *Z. mobilis* TISTR548 and *Z. mobilis Δgfo* under all conditions tested. Under heat, ethanol, and osmotic stresses, the specific growth rates of sorbitol-supplemented cultures of *Z. mobilis* TISTR548 were 1.81-, 1.42-, and 4.30-fold higher than those of the control cultures without sorbitol supplementation. A pronounced increase in cell growth under sorbitol supplementation was found in the *Z. mobilis* disruptant strain. The sorbitol-supplemented cultures of *Z. mobilis Δgfo* showed approximately 4.08-, 1.59-, and 6.19-fold higher in its specific growth rate than those of the control cultures without sorbitol supplementation under heat, ethanol, and osmotic stresses, respectively. With respect to the ethanol production, the maximum ethanol concentrations produced by sorbitol-supplemented cultures of *Z. mobilis* TISTR548 were 1.14-, 2.15-, and 2.14-fold higher than those of the control cultures without sorbitol supplementation under heat, ethanol, and osmotic stresses, respectively. In the case of *Z. mobilis Δgfo*, the maximum ethanol concentrations produced by sorbitol-supplemented cultures were 2.79-, 8.12-, and 42.49-fold higher than those of the control cultures without sorbitol supplementation under such stresses, respectively. These results clearly indicated that sorbitol reduced the sensitivity in growth and fermentation activity of cells to heat, ethanol and osmotic stresses.

**Table 3:** **Protective function of sorbitol on cell growth and ethanol production by *Z. mobilis *** **under heat, ethanol, and osmotic stresses**

**Treatment conditions**	**Fermentation parameter**					
	***Z. mobilis *** **TISTR548**			***Z. mobilis Δgfo***		
	***μ *** **(h**^**-1**^**)**	***P *** **(g L**^**-1**^**)**	***Q***_***P ***_**(g L**^**-1**^ **h**^**-1**^**)**	***μ *** **(h**^**-1**^**)**	***P *** **(g L**^**-1**^**)**	***Q***_***P ***_**(g L**^**-1**^ **h**^**-1**^**)**
*Heat stress*						
30°C	0.566±0.32	72.69±10.62	1.21±0.18	0.522±0.20	57.45±5.03	0.80±0.07
37°C	0.280±0.24	41.87±4.93	1.16±0.14	0.123±0.25	17.96±0.42	0.50±0.01
37°C + 50 mM sorbitol	0.508±0.30	47.70±3.56	1.33±0.17	0.502±0.22	50.12±1.54	1.04±0.06
*Ethanol stress*						
10% (v/v)	0.340±0.25	12.10 ± 3.20	0.25±0.12	0.286±0.18	3.10 ± 1.12	0.06 ± 0.01
10% (v/v) + 50 mM sorbitol	0.482±0.34	26.02 ± 2.15	0.54 ± 0.14	0.456±0.26	25.18 ± 2.45	0.52 ± 0.04
*Osmotic stress*						
300 g/L sucrose	0.126±0.32	39.76±2.21	0.66±0.04	0.084±0.24	1.62±0.01	0.03±0.01
300 g/L sucrose + 50 mM sorbitol	0.542±0.40	84.98±1.31	1.42±0.02	0.520±0.32	68.84±1.42	1.15±0.02

*μ*, specific growth rate (h^-1^); *P*, ethanol concentration produced (g L^-1^); *Q*_*p*_, volumetric ethanol productivity (g L^-1^ h^-1^).

### SDS-PAGE analysis

**Figure 4:**
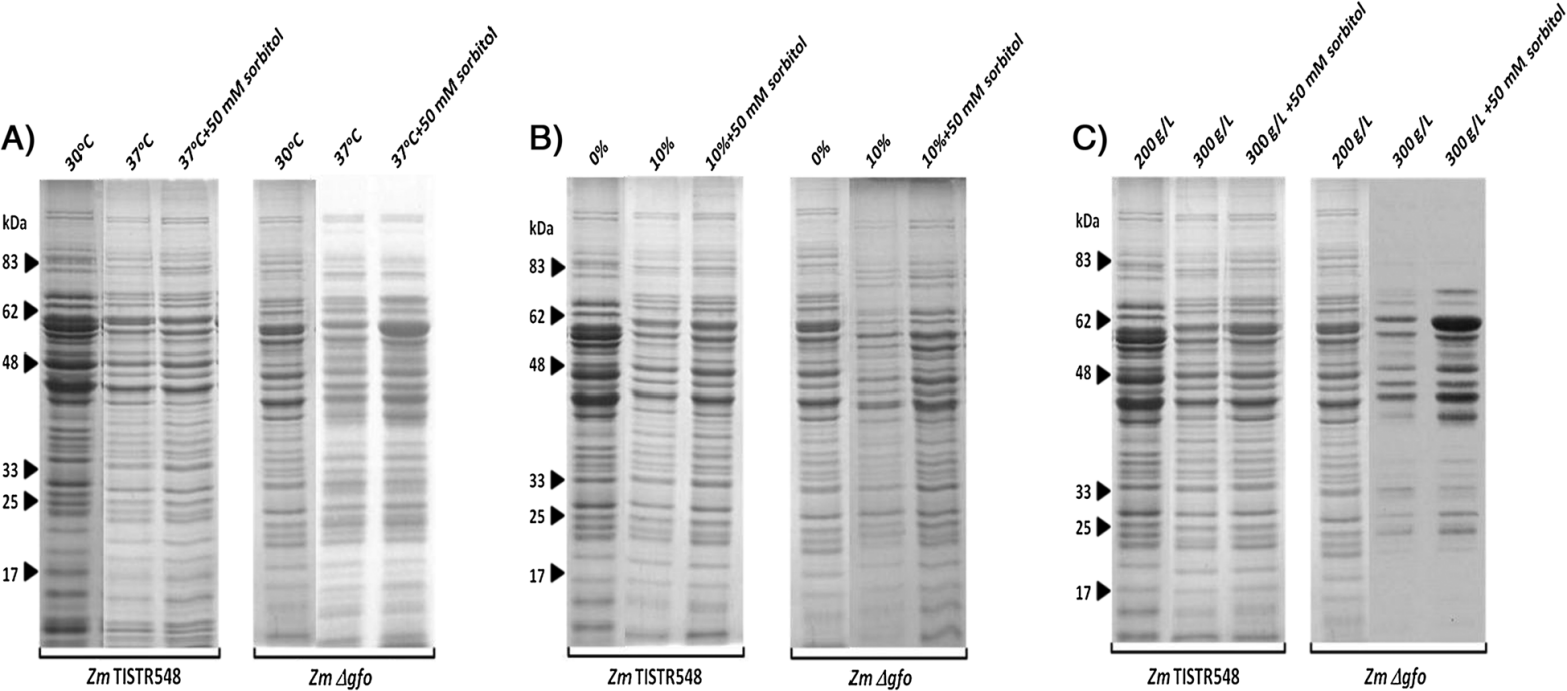
**SDS-PAGE analysis of protein isolated from *Z. mobilis *TISTR548 and *Z. mobilis Δgfo*.** Growing in medium supplemented with 50 mM sorbitol under **(A)** heat stress, **(B)** ethanol stress, and **(C)** osmotic stress.

The effect of heat, ethanol, and osmotic stresses on protein synthesis in *Z. mobilis* TISTR548 and *Z. mobilis Δgfo* were analyzed by SDS-PAGE and the results are shown in Figure 4. Under stress conditions, the protein synthesis in *Z. mobilis Δgfo* was almost suppressed as compared to that in *Z. mobilis* TISTR548 particularly under ethanol and osmotic stresses. Only a small number of proteins with the apparent molecular weight of approximately 58, 54, 48, 45, and 40 kDa were visualized in the protein sample isolated from *Z. mobilis Δgfo* growing under stress conditions. However, the synthesis of almost all proteins was recovered when sorbitol was supplemented into the culture medium. This can be seen from the protein patterns between sorbitol-supplemented and control cultures. In addition, the protein patterns from the sorbitol-supplemented cultures of *Z. mobilis Δgfo* were almost similar to those of *Z. mobilis* TISTR548, except the sorbitol-supplemented cultures of *Z. mobilis Δgfo* under osmotic stress. These results suggested that sorbitol may prevent protein from undergoing denaturation under heat, ethanol, and osmotic stresses.

### Complementation test of *Z. mobilis* disruptant strain

To determine whether the growth defect of the *Z. mobilis* disruptant strain under stress conditions is due to the deletion of *gfo* gene, the recombinant plasmid carrying the full length open reading frame (ORF) of the *gfo* gene was introduced into the disruptant strain, as described in the Methods section. A PCR product of approximately 1.3 kb was observed in the selected complemented strain, suggesting the presence of the full length ORF of the *gfo* gene in this strain (data not shown). Specific growth rate of the complemented strain under stress conditions was analyzed and the results are summarized in Table 4. Introduction of the full length ORF of the *gfo* gene rescued the growth defect of the *Z. mobilis* disruptant strain under heat, ethanol, and osmotic stresses. The complemented strain produced approximately 3.52 g/L of sorbitol when cultured in YP medium containing 300 g/L sucrose. These findings suggested that the *gfo* gene is required for proper growth of *Z. mobilis* under heat, ethanol, and osmotic stresses.

**Table 4:** **Specific growth rate of *Z. mobilis *** **parental, disruptant and complemented strains under heat, ethanol, and osmotic stresses**

**Stress conditions**	**Specific growth rate (h**^**-1**^**)**				
	**Parental**	**Parental (pZA22)**	**Disruptant**	**Disruptant (pZA22)**	**Complemented (pZA-GFORCOM)**
*Heat stress*					
30°C	0.568 ± 0.16	0.550 ± 0.17	0.520 ± 0.14	0.510 ± 0.13	0.564 ± 0.19
37°C	0.283 ± 0.12	0.271 ± 0.08	0.111 ± 0.04	0.102 ± 0.01	0.276 ± 0.11
*Ethanol stress*					
0% (v/v)	0.572 ± 0.18	0.568 ± 0.16	0.521 ± 0.18	0.506 ± 0.17	0.562 ± 0.17
10% (v/v)	0.329 ± 0.12	0.326 ± 0.11	0.291 ± 0.12	0.288 ± 0.11	0.321 ± 0.14
*Osmotic stress*					
200 gL^-1^	0.569 ± 0.22	0.562 ± 0.23	0.516 ± 0.18	0.511 ± 0.16	0.556 ± 0.21
300 gL^-1^	0.183 ± 0.10	0.177 ± 0.10	0.073 ± 0.06	0.075 ± 0.07	0.175 ± 0.09

## Discussion

During ethanol fermentation, ethanologenic microorganisms like *Z. mobilis* or *S. cerevisiae* may encounter several environmental stresses such as heat [12,14], ethanol [13,15], and osmotic stress at high sugar concentrations [11] and oxidative stress by endogenous reactive oxygen species (ROS) including hydrogen peroxide (H_2_O_2_) [16]. These stresses inhibit cell growth, cell division, cell viability [17,18], and the fermentation activity of cells [19]. They can also modify plasma membrane fluidity [20] as well as disrupt cellular ionic homeostasis, leading to a reduction of metabolic activity and eventually cell death. Therefore, defense mechanisms to overcome these stresses are important not only for the survival of these microorganisms but also for ethanol production. Synthesis of stress responsive proteins [12,21], fatty acids, particularly unsaturated fatty acids or saturated fatty acids, and ergosterol are a few of the mechanisms against heat and ethanol stresses which have been reported in *Z. mobilis* and *S. cerevisiae*[22]. In addition, formation of compatible solutes such as sorbitol in *Z. mobilis*[11] or trehalose in *S. cerevisiae*[23] to counteract the detrimental effects of osmotic stress, particularly under high sugar concentrations and ethanol stress, have also been reported.

Previous studies by Loos *et al*. [11] demonstrated that the addition of sorbitol into culture medium promoted cell growth of *Z. mobilis* in environments with high concentrations of sugar. However, to our knowledge, the protective function of sorbitol on cell growth and ethanol fermentation capability under heat and ethanol stresses in *Z. mobilis* has not been studied yet. The synthesis of GFOR protein is regulated by the *gfo* gene [24]. Therefore, the gene encoding GFOR (*gfo*) in *Z. mobilis* was disrupted in this study and the protective effects of sorbitol on cell growth and ethanol production in the *Z. mobilis* parental and disruptant strains were determined. Based on the fusion PCR-based construction technique, the *gfo* gene in *Z. mobilis* was disrupted, as confirmed by PCR analysis (Figure 1) and the determination of sorbitol content by HPLC in the parental and disruptant strains of *Z. mobilis* cells after cultivation in YP medium containing 300 g/L sucrose. This fusion PCR-based construction technique has been wildly used to disrupt many genes, (for example cytochrome c peroxidase gene (*cytC*) in *Z. mobilis*[25]).

As shown in this study, disruption of the *Z. mobilis gfo* gene resulted in the reduction of cell growth both on solid and in liquid medium particularly under heat, ethanol, and osmotic stresses. The morphology of *Z. mobilis* parental and disruptant strains grown under stress conditions was not different after the microscopic analysis, suggesting that the disruption of the *gfo* gene did not interfere with cell morphology in this organism (data not shown). As shown in Table 1, the specific growth rates of the *Z. mobilis* disruptant strain grown under stress conditions were less than those of the *Z. mobilis* parental strain. However, when sorbitol was supplemented into the culture medium, the specific growth rates of *Z. mobilis* parental and disruptant strains grown under heat, ethanol and osmotic stresses were increased as compared to the conditions without sorbitol supplementation. Complementation experiments by introducing the full length ORF of the *gfo* gene into the *Z. mobilis* disruptant strain rescued the growth defect under heat, ethanol, and osmotic stresses (Table 4). These results clearly indicated that sorbitol is required for proper cell growth not only under osmotic stress as demonstrated by Loos *et al*. [11], but also under heat and ethanol stresses. We speculate from these findings that sorbitol may play a crucial role in cellular protection, by stabilizing the membrane bilayer or involving membrane fluidity, and by recovery of the cells from the stress.

Under stress conditions, the ethanol production capability of *Z. mobilis* parental and disruptant strains, as measured by ethanol concentration, volumetric ethanol productivity and ethanol yield, was decreased as compared to that under control conditions. Moreover, the ethanol fermentation ability of the *Z. mobilis* disruptant strain was also lower than that of the *Z. mobilis* parental strain (Table 2). This finding clearly demonstrated that heat, ethanol, and osmotic stresses caused inhibition of ethanol fermentation in *Z. mobilis*. One possible explanation that has been proposed for such detrimental effects is that heat, ethanol, and osmotic stresses cause a reduction in the effectiveness of the plasma membranes as a semipermeable barrier and transport process, allowing leakage of essential cofactors and coenzymes required for the activity of enzymes involved in glucose catabolism and alcohol production [19,26]. Surprisingly, the ethanol fermentation activity of *Z. mobilis* parental and disruptant strains was recovered when sorbitol was added into the fermentation medium (Table 3). This finding suggested that sorbitol not only enhanced cell growth but also increased the fermentation ability of this organism under stress conditions, since the expression levels of *pdc*, *adhA*, and *adhB* genes involved in the PE pathway in the *Z. mobilis* disruptant strain was lower than that in the parental strain (Figure 3). Thus, reduction in the ethanol fermentation ability of the *Z. mobilis* disruptant strain might be due to the suppression of these genes by heat, ethanol, and osmotic stresses.

We have attempted to investigate the effect of heat, ethanol, and osmotic stresses on protein synthesis in *Z. mobilis* parental and disruptant strains growing under stress conditions. As shown in Figure 4, the protein levels (as determined by protein intensity in the gel) of *Z. mobilis* disruptant strain growing under heat, ethanol, and osmotic stresses were lower than those of the *Z. mobilis* parental strain. One possibility is that heat, ethanol, and osmotic stresses suppress the synthesis of protein as well as cause protein denaturation [26]. These results are in agreement with those reported by Chandler *et al*. [27] and Hu *et al*. [28]. The synthesis of protein was increased when sorbitol was supplemented into the culture medium particularly proteins with the molecular weight of approximately 58, 54, 48, 45, and 40 kDa. We speculate from this finding that sorbitol plays a crucial role in the protection of proteins under heat, ethanol, and osmotic stresses. Yoo and Lee [29] reported that sorbitol can be used to preserve protein during storage since it can protect protein during dehydration by heat stress.

Based on these findings, we considered that sorbitol not only promoted cell growth but also increased the ethanol fermentation capability of *Z. mobilis* under heat, ethanol, and osmotic stresses. Increasing cell growth and ethanol fermentation activity in *Z. mobilis* might be related to the stabilization of cellular proteins by sorbitol. We are currently focused on the characterization of the genes and proteins responsible for sorbitol supplementation under stress conditions.

With respect to the substrate utilization, the narrow substrate spectrum with only three sugars, glucose, fructose, and sucrose, makes *Z. mobilis* not suitable for ethanol fermentation from sugar- and sugar-based feedstocks. On the other hand, ethanol yield from fructose and sucrose is very low since this microorganism accumulates sorbitol as a major byproduct. If *Z. mobilis* is engineered with pentose metabolic pathways, it might be suitable for ethanol production from lignocellulose biomass, since glucose is the only monomer sugar released from cellulose hydrolysis. The unique byproduct sorbitol might also provide some protection on cells from the toxicity of byproducts released during the pretreatment process of lignocellulose biomass. This hypothesis is now under our investigation.

## Conclusions

*Z. mobilis Δgfo* required the compatible solute, sorbitol, for normal cell growth, ethanol production as well as synthesis of cellular protein under stress conditions including heat, ethanol and osmotic stresses. This finding suggested that sorbitol plays an important role in the process of cell growth, ethanol fermentation, and protein synthesis not only under osmotic stress as previously reported but also under heat and ethanol stresses. Supplementation of sorbitol into the culture medium may be one of the effective approaches to improve the production yield of bioethanol or other chemicals under stress conditions with high temperature, high ethanol, or high sugar concentrations.

## Methods

### Strains, plasmid, and culture conditions

*Z. mobilis* TISTR548 was obtained from the Thailand Institute of Scientific and Technological Research (TISTR), Bangkok for use in this study. This strain exhibited higher growth and ethanol fermentation ability at relatively high temperature fermentation conditions (37°C and 40°C) than those of the type strain, *Z. mobilis* ZM4 [30]. Both *Z. mobilis* TISTR548 and the *gfo* disruptant strain, *Z. mobilis Δgfo*, were grown in YPG medium (3 g/L yeast extract, 5 g/L peptone, 30 g/L glucose) at 30°C [31]. When necessary, sugar stock solution autoclaved separately was added. Cultures were maintained on YPG medium solidified with 2% agar at 4°C with subculturing every 2 months. For the extraction of nucleic acids, *Z. mobilis* cells were grown in YPG medium at 30°C on a rotary shaker (100 rpm). After 16 hours of incubation, cells were harvested by centrifugation at 5,000 rpm for 5 minutes and washed twice with sterilized distilled water. *Escherichia coli* strain DH5α was used for gene manipulation. It was grown in Luria-Bertani (LB) medium at 37°C on a rotary shaker (100 rpm). Shuttle vector pZA22, kindly provided by Professor Hideshi Yanase, was used to clone DNA fragments and to disrupt the *Z. mobilis gfo* gene in fusion PCR-based construction experiments. This vector contains the chloramphenicol (*cm*) and tetracycline (*tc*) resistant marker genes.

### DNA isolation and disruption of the *Z. mobilis* gfo gene

**Figure 5:**
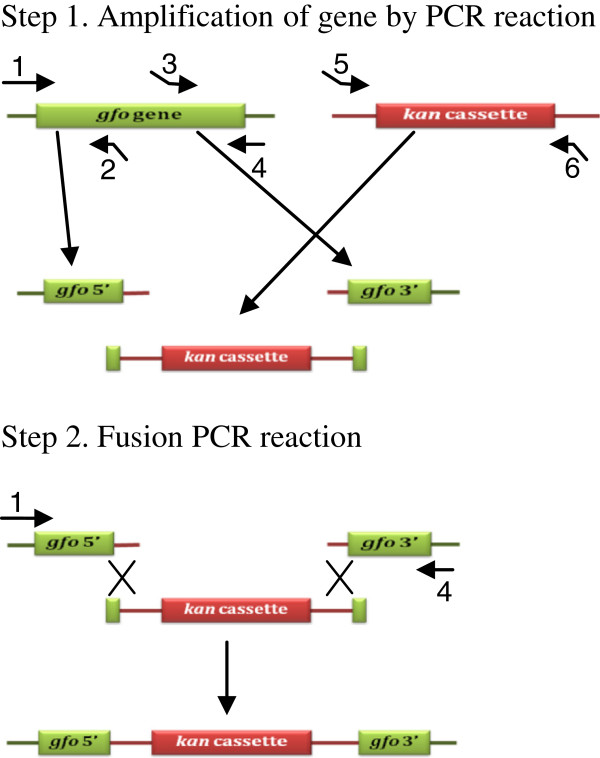
**Schematic illustration of PCR-based construction of the *gfo *** **gene disruption.**

The genomic DNA of *Z. mobilis* was prepared by the standard method [32]. The *gfo* gene in *Z. mobilis* was disrupted by the fusion PCR-based construction technique [33]. The procedures for gene disruption are illustrated in Figure 5 and the primers used in the gene disruption experiment are shown in Table 5. In the first step, the 5’- and 3’-flanking regions of the *gfo* gene were amplified by PCR using specific primers, GFOR-up-5’, GFOR-up-kan-3’, GFOR-down-kan-5’, and GFOR-down-3’, synthesized based on the *gfo* gene in *Z. mobilis*. The specific primers, GFOR-up-kan-3’ and GFOR-down-kan-5’, directly adjacent to the marker cassette contain 5’-end (primer GFOR-up-kan-3’) and 3’-end (primer GFOR-down-kan-5’) sequences of *kan* cassette at their 5’-ends. Two specific primers, kan-GFOR-5’ and kan-GFOR-3’, which sequences complementary to primer GFOR-up-kan-3’ and GFOR-down-kan-5’ were used to amplify the *kan* cassette. In the second step, the 5’- and 3’-flanking regions of the *gfo* gene were joined to the DNA fragment of the *kan* cassette by PCR using the outermost primer GFOR-up-5’ and GFOR-down-3’, and the resulting DNA fragment of approximately 4.2 kb in length was cloned into pGEM-T Easy Vector (Promega, Medison, WI, USA) and then transformed into *E. coli* DH5α by electroporation. All PCR reactions were performed by using a PCR amplification kit (Takara Biomedical, Otsu, Japan) according to the manufacturer’s instructions. The positive clone was selected based on an ampicillin resistance marker. Plasmid DNA was isolated from this clone using the standard method [32] and then subjected to nucleotide sequencing using a DNA sequencing kit (Applied Biosystems, Foster City, CA, USA) with a MegaBACE 1000 automated DNA sequencer (Pharmacia Biotech, Uppsala, Sweden). After *Eco*RI digestion, a DNA fragment containing the up- and down-region of the *gfo* gene linked with *kan* cassette was ligated into the *Eco*RI site of shutter vector pZA22 to generate plasmid pZA-UDGFOR. This plasmid was then transformed into *Z. mobilis* by electroporation technique. After transformation, the transformants were screened based on the kanamycin resistance marker, and the *gfo* gene in the selected transformants was confirmed by PCR using primers GFOR-int-5’ and GFOR-int-3’ synthesized based on an internal fragment of the *gfo* gene in *Z. mobilis*, with genomic DNA isolated from the parental strain and transformants as template. Nucleotide sequencing of the PCR product was carried out as previously described to confirm the amplified product. Online database comparisons were performed with the BLAST algorithm in the GenBank and DNA DataBank of Japan (DDBJ) databases. To further confirm the disruption of the *gfo* gene, sorbitol production by *Z. mobilis* parental strain and disruptant was measured after growing both strains in YP medium containing 300 g/L sucrose as a carbon source.

**Table 5:** List of primers used in the study

**Gene name**	**Primer name**	**Sequences (5’ to 3’)**	**Usage**
*gfo*	GFOR-up-5’	TGCCGCAGATAAACTCG	Gene disruption
	GFOR-up-kan-3’	CAGCTCCAGCCTACACAGATTATCTGAAGACGAGA	
	GFOR-down-kan-5’	AAGGAGGATATTCATATGTATGAAGCAGCTCGTACC	
	GFOR-down-3’	CGCAAGAACCAATACCG	
	GFOR-int-5’	AACGGATGACAACGCTT	
	GFOR-int-3’	AGTCGTCGTGGTCGAAT	
*kan*	kan-GFOR-5’	TCTCGTCTTGAGATAATCTGTGTAGGCTGGAGCTG	
	kan-GFOR-3’	GGTACGAGCTGCTTCATACATATGAATATCCTCCTT	
*adhA*	adhA-F	CATGAAAGCAGCCGTCA	Real-time PCR
	adhA-R	TACACCCGCGCAAGTGA	
*adhB*	adhB-F	GTCAACGAAATGGGCGA	
	adhB-R	GTGACGGTCAACAATGG	
*pdc*	pdc-F	GACTACAACCTCGTCCT	
	pdc-R	CAGGGCATGGGAGCAAT	
*16 s RNA*	16 s-F	CAGCACCTGTCTCTGATCCA	
	16 s-R	GTTCGGAATTACTGGGCGTA	
*gfo*^*a*^	GFOR-com-5’	GC*GAATTC*ATGACGAACAAAATCTCG	Complementation
	GFOR-com-3’	GC*GAATTC*TCAATAACCACCCTGACG	

^a^The italic sequence corresponds to the *Eco*RI site.

### Effect of stresses on the growth of *Z. mobilis* parental and disruptant strains

The effect of stresses including heat, ethanol, and osmotic stresses on the growth of *Z. mobilis* parental and disruptant strains were tested. For heat stress, cells were grown on YP agar medium (3 g/L yeast extract, 5 g/L peptone) containing 200 g/L sucrose and incubated at 30, 35, 37, and 39°C for 2 days. For ethanol stress, cells were grown on YP agar medium containing 200 g/L sucrose and ethanol at a final concentration of 7, 10, and 13% (v/v), and then incubated at 30°C for 2 days. The effect of osmotic stress on cell growth was determined by culturing cells on YP agar medium containing 200, 250, and 300 g/L sucrose for 2 days at 30°C. After 2 days of cultivation, a part of culture was taken and serial dilutions of 10 times were made, and 10 μL of each dilution was spotted on YP agar plate [34].

The effect of stresses on the specific growth rate of *Z. mobilis* parental and disruptant strains was also compared. For heat stress, cells were grown in YP liquid medium containing 200 g/L sucrose and then incubated at 30, 35, 37, and 39°C. For ethanol stress, cells were grown in liquid YP medium containing 200 g/L sucrose and ethanol at a final concentration of 0, 7, 10, and 13% (v/v), and then incubated at 30°C. For osmotic stress, cells were grown in liquid YP medium containing sucrose at a final concentration of 200, 250, and 300 g/L and incubated at 30°C. During cultivation, cells were harvested every 2 hours and spread onto YP agar plate. Cell growth was determined by colony-forming units (CFUs) and the specific growth rate of bacterial cells was calculated as described by Keeratirakha [35]. All experiments were performed in quadruplets and repeated twice. The data are means (±SD) of the results of the experiments.

### Effect of stresses on ethanol fermentation by *Z. mobilis* parental and disruptant strains

Batch ethanol fermentation by *Z. mobilis* parental and disruptant strains was carried out in 500 mL Erlenmeyer flasks equipped with an air-lock for an anaerobic growth condition as described by Taherzadeh et al. [36]. Next, 10% of active *Z. mobilis* cells were inoculated into 400 mL YP liquid medium containing sucrose as a sole carbon source and statically incubated at 30°C. The influence of incubation temperatures on ethanol fermentation by *Z. mobilis* was performed by culturing cells in liquid YP medium containing 200 g/L sucrose and incubated at various temperatures (30, 35, and 37°C). The effect of ethanol stress on ethanol fermentation by *Z. mobilis* was determined by culturing cells in liquid YP medium containing 200 g/L sucrose and ethanol at different concentrations (0, 7.0, and 10%) and then incubated at 30°C. Likewise, the effect of osmotic stress on ethanol production was evaluated by culturing cells in liquid YP medium containing different sucrose concentrations (200, 250, and 300 g/L) and incubated at 30°C.

The protective effect of sorbitol against heat, ethanol, and osmotic stresses was tested. Briefly, the cultures growing in liquid YP medium containing 200 g/L sucrose were exposed to heat (37°C) and ethanol (10%) stresses in the presence of 50 mM sorbitol. For osmotic stress, cultures were grown in liquid YP medium containing 300 g/L sucrose in the presence of 50 mM sorbitol. During ethanol fermentation, samples were periodically withdrawn and analyzed for cell growth (as specific growth rate) and ethanol content. All the fermentation experiments were replicated twice and the average values ±SD are presented in this study.

### RT-qPCR analysis of gene expression under stresses

The expressions of alcohol dehydrogenase A (*adhA*), alcohol dehydrogenase B (*ahdB*), and pyruvate decarboxylase (*pdc*) genes in *Z. mobilis* parental and disruptant strains were determined by real-time RT-PCR. Total RNA was isolated from *Z. mobilis* parental and disruptant strains grown in YP liquid medium under stress conditions, as described above, by using the GF-1 Total RNA Extraction Kit (Vivantis, Eco-Life Science, Kowloon, Hong Kong). The concentration of RNA was measured and adjusted by Nanodrop (Nanodrop Technologies, Wilmington, DE, USA). First-strand cDNA synthesis and SYBR Green RT-PCR assays were performed according to the manufacturer’s instruction. The real-time RT-PCR amplifications were performed using the CFX96 Touch Real-Time PCR Detection System (Bio-Rad, Hercules, CA, USA). The reactions were carried out in a total volume of 25 μL containing 12.5 μL iQ SYBR Green Supermix, 0.5 μL diluted cDNA, 11.0 μL sterile water, and 0.5 μL of each forward and reverse primer. The thermal cycling conditions for PCR were initial denaturation at 95°C for 3 minutes, followed by 40 cycles each of denaturation at 95°C for 10 seconds, and annealing at 55°C for 30 seconds. The expression of 16 s RNA was analyzed as an internal control with specific primer as shown in Table 5. As a negative control, DEPC-treated water was used instead of cDNA template. All experiments were independently repeated at least twice in order to ensure reproducibility of the results.

Data from real-time RT-PCR amplifications were analyzed using CFX Manager Software (Bio-Rad, USA). The comparative *Ct* method was used to analyze the expression levels of *adhA*, *adhB*, and *pdc* genes, based on the method described by Jiang et al. [37]. For data analysis, the relative expression levels were imported into Microsoft Excel (Redmond, WA, USA) for subsequent statistical analysis. All data are presented as the mean of relative mRNA expression.

### Protein extraction and SDS-PAGE

Heat-, ethanol-, and osmotic-stressed cells were harvested by centrifugation, washed with sterilized distilled water, and suspended in a 10 mM Tris HCl (pH 7.0) buffer. Proteins were extracted from stressed cells by sonicating in Bioruptor (Cosmo Bio, Tokyo, Japan) for 10 minutes at 50% pulse duty cycle with the output power of 5, as described by Thanonkeo et al. [12]. Protein concentration of the cell-free extracts was measured using Lowry reagent. For electrophoresis, approximately 20 μg of the protein sample was heated at 100°C for 5 minutes and separated by SDS electrophoresis on 12% acrylamide gel with a constant voltage of 50 V. After electrophoresis, proteins separated on the gel were visualized using Coomassie Brilliant Blue R250 and fixed in 10% ethanol.

### Complementation experiment

The full length ORF of the *gfo* gene was amplified by PCR with genomic DNA isolated from *Z. mobilis* parental strain as a template and specific primers, GFOR-com-5’ and GFOR-com-3’ (Table 5), synthesized based on the 5’- and 3’-region of the *gfo* gene in this organism*.* All PCR reactions were performed by using a PCR amplification kit (Takara Biomedical) according to the manufacturer’s instruction. The amplified product was purified and ligated into the *Eco*RI site of the shutter vector, pZA22. The resultant recombinant plasmid was designated as pZA-GFORCOM. This plasmid was then transformed into *Z. mobilis* disruptant strain by electroporation. After transformation, the complemented cells were screened based on the tetracycline resistance marker and confirmed by checking growth ability under stress conditions. The specific growth rate of the selected complemented strain grown in YP liquid medium under heat (37°C), ethanol (10% v/v), and osmotic stress (300 g/L sucrose) was determined. The production of sorbitol in YP medium containing 300 g/L sucrose by complemented strain was also examined. In addition, the replacement of the *kan* cassette by the full length ORF of the *gfo* gene after homologous recombination in the selected complemented strain was confirmed by PCR using specific primers GFOR-com-5’ and GFOR-com-3’, as described above. The *Z. mobilis* parental and disruptant strains carrying pZA22 were generated as a control.

### Analytical methods

The fermentation broths were centrifuged at 13,000 rpm for 10 minutes to remove cells. The supernatant was then determined for total residual sugars by phenol sulfuric acid method [38]. Sorbitol concentration was analyzed by HPLC using an Aminex HPX 87C column (300 mm × 7.8 mm, temperature 80°C) with a refraction index detector. The distilled deionized water was used as a mobile phase at a flow rate of 0.6 mL/min and sorbitol was used as a standard. Ethanol concentration (*P*, g/L) was analyzed by gas chromatography (GC) (GC-14B, Shimadzu, Kyoto, Japan) using polyethylene glycol (PEG-20 M) packed column, nitrogen as a carrier gas, and a flame ionization detector (FID). Ethanol was quantified by using 2-propanol as an internal standard [39]. The ethanol yield (*Yps*) was calculated as the actual ethanol produced and expressed as gram ethanol per gram sugar utilized (g/g). The volumetric ethanol productivity (*Qp*, g/L.h) was calculated by the following equations: *Qp* = *P/t* ; where *P* is the ethanol concentration (g/L) and *t* is the fermentation time (hours) giving the highest ethanol concentration.

## Abbreviations

adhA: Alcohol dehydrogenase A; adhB: Alcohol dehydrogenase B; BLAST: Basic Local Alignment Search Tool; CFU: Conoly-forming unit; DDBJ: DNA DataBank of Japan; DEPC: Diethylpyrocarbonate; ED: Entner-Doudoroff; EMP: Embden-Meyerhof-Parnas; FID: Flame ionization detector; GC: Gas chromatography; GFOR: Glucose-fructose oxidoreductase; GP: Glyceraldehydes 3-phosphate-to-pyruvate; GRAS: Generally recognized as safe; HPLC: High performance liquid chromatography; KDPG: 2-keto-3-deoxy-6-phosphogluconate; LB: Luria-Bertani; ORF: Open reading frame; PCR: Polymerase chain reaction; pdc: Pyruvate decarboxylase; PE: Pyruvate-to-ethanol; PEG: Polyethylene glycol; ROS: Reactive oxygen species; RT: Reverse transcriptase; TCA: Tricarboxylic acid; TISTR: Thailand Institute of Scientific and Technological Research; YP: Yeast extract peptone; YPG: Yeast extract peptone glucose.

## Competing interests

The authors declare that they have no competing interests.

## Authors’ contributions

KS carried out the disruption of the *gfo* gene in *Z. mobilis*. PT participated in the design of the study, carried out the expression analysis of genes and proteins and, complementation test, and wrote the manuscript. NK carried out the research experiments and data analysis. ST and PJ participated in the design of the study. MY participated in the design of the study and helped to draft the manuscript. All authors read and approved the final manuscript.
